# Untargeted Urinary Volatilomics Reveals Hexadecanal as a Potential Biomarker for Preeclampsia

**DOI:** 10.3390/ijms252212371

**Published:** 2024-11-18

**Authors:** Marina Pehlić, Stipe Dumančić, Mila Radan, Jelena Galić, Branimir Gruica, Sandra Marijan, Marko Vulić

**Affiliations:** 1 Department of Gynecology and Obstetrics, University Hospital of Split, 21000 Split, Croatia; marinapehlic82@gmail.com (M.P.); stipe.dumancic@gmail.com (S.D.); mvulic67@gmail.com (M.V.); 2 Department of Biochemistry, Faculty of Chemistry and Technology, University of Split, 21000 Split, Croatia; jg19038@ktf-split.hr (J.G.); bg19046@ktf-split.hr (B.G.); 3 Department of Medical Chemistry and Biochemistry, University of Split School of Medicine, 21000 Split, Croatia; sandra.marijan@mefst.hr

**Keywords:** preeclampsia, oxidative stress, VOCs (volatile organic compounds), sphingolipids

## Abstract

Preeclampsia (PE) is a severe hypertensive pregnancy disorder characterized by endothelial dysfunction, placental ischemia and oxidative stress; however, reliable non-invasive biomarkers for early detection are limited. In this study, untargeted solid-phase microextraction with gas chromatography–mass spectrometry (SPME-GC-MS) was used to analyze volatile organic compounds in the urine of 45 women with PE and 46 healthy controls. Among the 29 metabolites identified, hexadecanal—a product of lipid peroxidation and sphingolipid metabolism—was found to be the most significant, with an area under the receiver operating characteristic (ROC) curve of 0.618, highlighting its diagnostic potential. This result emphasizes the role of hexadecanal in oxidative stress and placental dysfunction, which are central to the pathophysiology of PE. The results support hexadecanal as a potential non-invasive biomarker while demonstrating the efficacy of SPME-GC-MS in identifying metabolic disorders associated with PE, paving the way for further research to confirm its clinical utility for early diagnosis and risk assessment.

## 1. Introduction

Preeclampsia is a complex multisystem disease, diagnosed by the sudden onset of hypertension (>20 weeks of gestation) and at least one other associated complication, including proteinuria, maternal organ dysfunction or uteroplacental dysfunction [1]. The pathogenesis of PE is complex and involves abnormal placentation, leading to altered uteroplacental perfusion characterized by ischemia–reperfusion injury to the syncytiotrophoblast, which ultimately leads to placental dysfunction. This ischemia leads to oxidative stress (OS) in the placenta, which triggers a cascade of systemic inflammatory responses in the mother characterized by endothelial dysfunction. These processes share similarities with other pregnancy complications, including fetal growth restriction (FGR) and miscarriage [2,3]. Oxidative stress-induced endothelial dysfunction is a key factor in the progression of PE and contributes to maternal symptoms such as hypertension and proteinuria. The imbalance between pro- and anti-angiogenic factors, especially the increased levels of soluble FMS-like tyrosine kinase-1 (sFlt-1) and decreased placental growth factor (PlGF), further exacerbate the condition by impairing vascular remodeling and blood flow in the placenta [4].

Oxidative stress reflects a disturbance in the balance between reactive oxygen species (ROS) and the body’s antioxidant defenses. While normal pregnancies have low levels of oxidative stress, PE is associated with a significant reduction in antioxidant capacity, resulting in increased OS [5]. A recent meta-analysis identified several biomarkers of oxidative stress, including ischemia-modified albumin (IMA), uric acid (UA) and malondialdehyde (MDA), which are useful for the early detection of PE [2]. These findings emphasize the critical role of oxidative stress in the pathophysiology of PE and the importance of identifying reliable biomarkers for early diagnosis and intervention.

Metabolomics has become a powerful tool for studying the comprehensive metabolic profile of biological systems, providing insights into the biochemical changes associated with disease states [6]. In particular, untargeted metabolomics allows for an unbiased analysis of metabolites without prior assumptions about the metabolic profile of the sample [7]. This approach has contributed to the identification of potential biomarkers for various diseases, including pregnancy-related disorders such as PE [8]. Advanced analytical techniques such as nuclear magnetic resonance (NMR) and mass spectrometry (MS) are frequently used in metabolomics [9]. While NMR offers high reproducibility and minimal sample preparation, MS is characterized by its higher sensitivity and specificity, making it the preferred method for metabolomic studies in complex biological samples [10].

Hexadecanal (palmitaldehyde, C_16_H_32_O) is a long-chain fatty aldehyde that is involved in the biosynthesis of sphingolipids and fatty alcohols, essential components of cell membranes [11]. Hexadecanal has been detected not only in human biological systems but also in a variety of other species, including fungi (Saccharomyces cerevisiae), mice and plants. Hexadecanal is a reactive carbonyl species formed by the lipid peroxidation of polyunsaturated fatty acids (PUFAs) in triacylglycerols, phospholipids and cholesteryl esters [12]. Oxidative stress-induced lipid peroxidation has been linked to numerous pathological conditions, including inflammation, atherosclerosis, neurodegenerative diseases and cancer. Although hexadecanal has been less studied than other lipid peroxidation products, such as 4-hydroxy-2-nonenal (HNE) and malondialdehyde (MDA), its role in oxidative damage makes it a potentially important biomarker for PE [13].

Due to its high sensitivity and reproducibility, gas chromatography–mass spectrometry (GC-MS) is a widely used technique in metabolomics for the analysis of volatile organic compounds (VOCs) [14]. Despite its advantages, conventional GC-MS often requires extensive sample preparation, including solvent extraction and chemical derivatization, which can be time-consuming and costly [15]. However, with the advent of solid-phase microextraction (SPME), the procedure was simplified, especially when analyzing biofluids such as urine [15,16]. SPME is a solvent-free, efficient extraction method that uses a fiber coated with an extraction phase to capture low-molecular-weight metabolites, which are then thermally desorbed and introduced into the GC-MS system for analysis. This technique has proven particularly useful in the analysis of volatiles due to its reproducibility, cost efficiency and versatility. The ability of SPME to extract volatile compounds without the need for complex sample preparation makes it an attractive option for metabolomic studies of urine [17].

Urinary volatilomics remains an under-researched area in PE research, although its potential to identify new non-invasive biomarkers is increasingly recognized. Urinary volatile metabolites may offer valuable insights into the biochemical disturbances associated with PE, particularly in relation to oxidative stress and consequently lipid peroxidation. In a recent study, Sana et al. used SPME-GC-MS to investigate the metabolite profiles of women with gestational diabetes mellitus (GDM) and identified several volatile metabolites in both urine and serum [18]. However, there is a paucity of data on urinary metabolomics in PE, highlighting the need for further research in this area.

Therefore, the aim of this study is to investigate the urinary metabolite profiles of women with PE compared to healthy pregnant women using non-targeted SPME-GC-MS analysis. By identifying volatile organic compounds associated with PE, this study aims to contribute to the growing body of knowledge on biomarkers related to oxidative stress and to improve the understanding of metabolic pathways involved in the pathophysiology of PE.

## 2. Results

### 2.1. Baseline Clinical Characteristics of Enrolled Participants

This study included 45 patients with PE and 46 patients without PE, who were included in the control group (Table 1). The age distribution of the patients was similar, with an average age of about 32 years, with no statistical differences. However, patients with PE had significantly lower parity compared to the control group, especially primipara (*p* = 0.003). In addition, patients with PE had a higher pre-pregnancy body mass index (BMI) compared to the control group (median 26.35 kg/m^2^ versus 24.51 kg/m^2^, *p* = 0.016). There was no statistical difference in weight gain during pregnancy.

PE is considered a perinatal complication with an elevated arterial blood pressure of over 140/90 and the detection of proteins (albumins) in the urine, which were the diagnostic criteria and inclusion criteria for the participants in the PE group. The inclusion criteria for participants in the control group were patients with pregnancies without pregestational comorbidities or metabolic complications in pregnancy, including patients with breech presentation of the fetus, cephalic disproportion without the detection of gestational diabetes mellitus, previous history of cesarean delivery or myomectomy and uterine inertia.

Exclusion criteria were pregnancies with metabolic complications during pregnancy such as gestational diabetes, premature labor, urinary tract infections, intrahepatic cholestasis during pregnancy and liver disease, Rh immunization, chorioamnionitis and infections (TORCH syndrome, parvo-B19 virus, hepatitis and COVID-19 infection). In addition, patients with concomitant prenatal diseases, such as polycystic ovary syndrome, diabetes mellitus, autoimmune diseases, chronic neurological diseases, malignant diseases and patients with chronic therapy, were excluded.

### 2.2. Identified Volatile Metabolites in the Analyzed Urine Samples

A metabolomic analysis of volatile organic compounds was performed on 91 urine samples consisting of 45 samples from women diagnosed with PE and 46 control samples. The analysis was performed using GC-MS with headspace solid-phase microextraction (HS-SPME), which led to the identification of a distinct metabolomic profile. In this study, 29 volatile organic metabolites out of a total of 107 detected compounds were identified in all urine samples of PE and control groups. GC-MS with HS-SPME analysis provided a detailed profile of the volatile organic metabolites present. The identified compounds are listed in Table 2.

### 2.3. Univariate Statistical Analysis

Univariate statistical analysis was used to determine significant differences in metabolite levels between the control group (C) and the preeclamptic group (PE).

**Figure 1:**
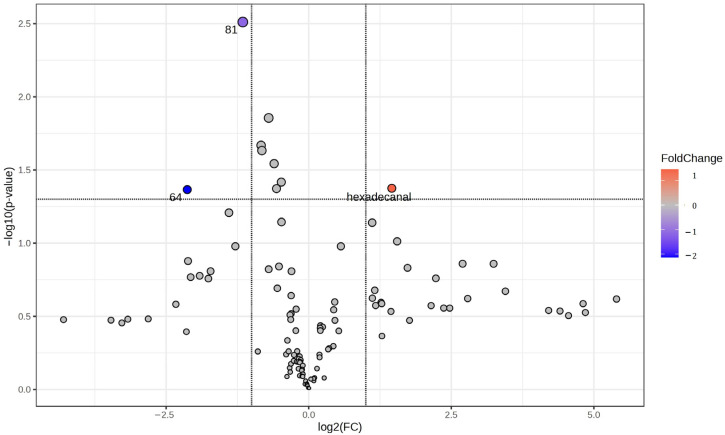
Volcano plot highlighting statistically significant metabolites in the preeclampsia vs. control groups.

Table 3 shows the metabolites with significant fold-change values and *p*-values. Among them, hexadecanal showed the largest change, indicating its potential as an important biomarker for PE (Figure 1).

### 2.4. Biomarker Analysis

**Figure 2:**
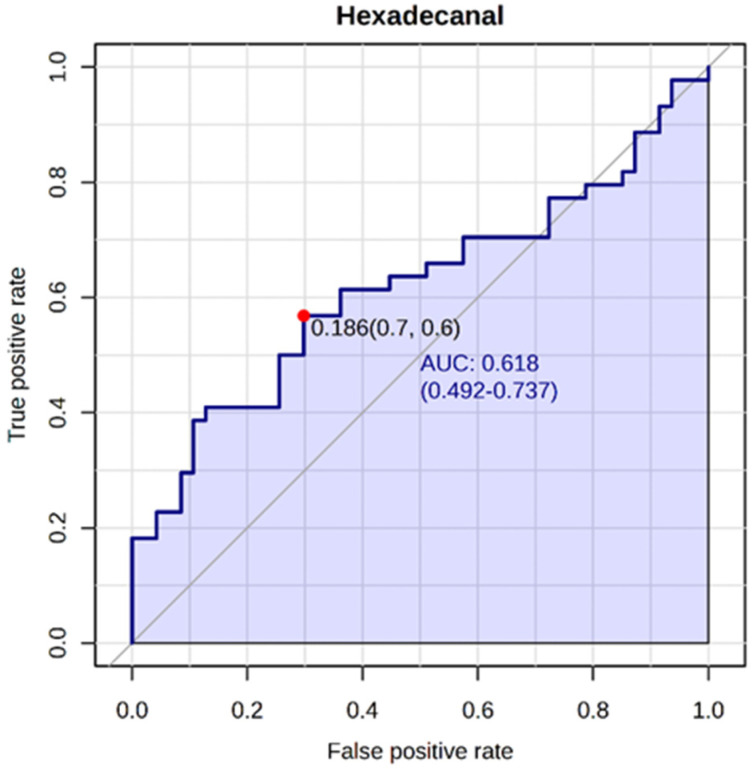
ROC curve for hexadecanal.

Based on the statistical analyses, hexadecanal proved to be the most significant metabolite with a high potential as a biomarker for PE. To further evaluate its diagnostic value, a receiver operating characteristic (ROC) curve was generated (Figure 2), which provides insight into its ability to discriminate between the PE and control groups by balancing sensitivity (true positive ratio) and specificity (true negative ratio). The area under the curve (AUC) for hexadecanal was calculated to be 0.618 (95% CI: 0.492–0.737), indicating a moderate diagnostic ability. The sensitivity was 0.6 (60%) and the specificity was 0.7 (70%), reflecting reasonable diagnostic performance. Although the AUC does not indicate exceptional predictive accuracy, hexadecanal shows promising potential as a biomarker for PE that warrants further investigation.

### 2.5. Enrichment Analysis

**Figure 3:**
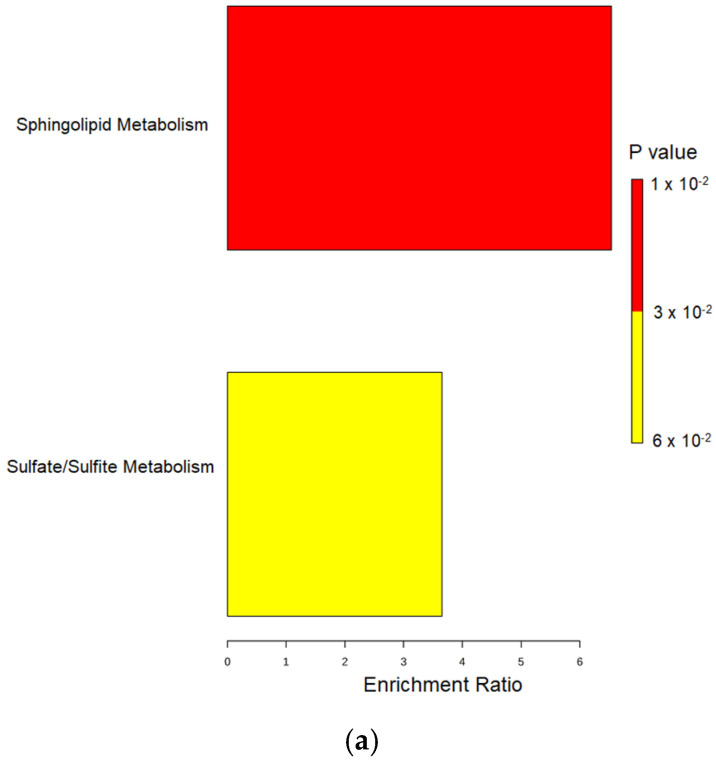

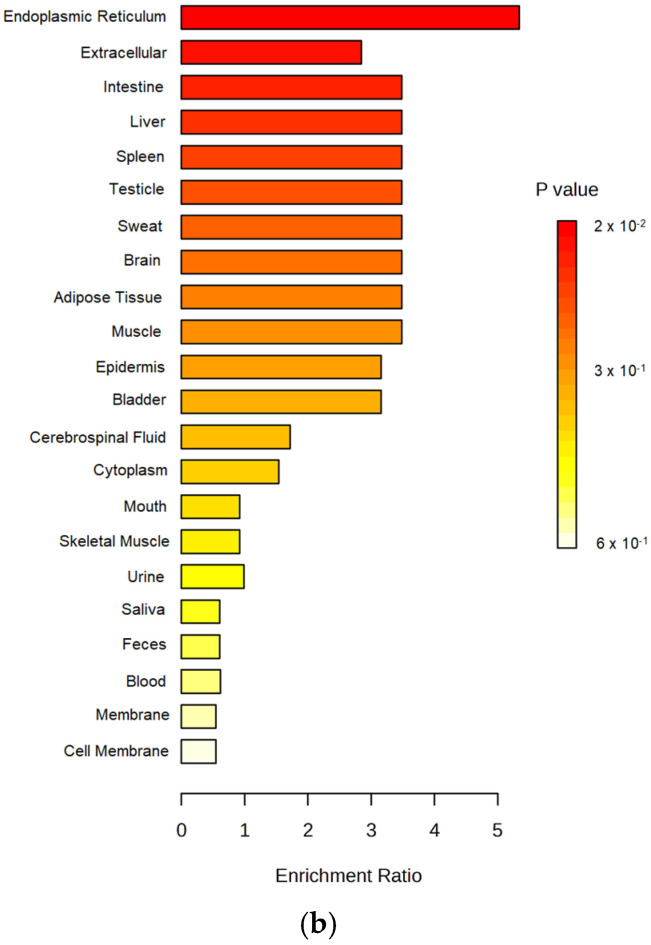
Metabolomic set enrichment analysis identifying key pathways and (**a**) cellular locations in preeclampsia (**b**).

Enrichment analysis is a statistical method for identifying metabolic pathways and metabolic groups of metabolites that are particularly important in a specific biological context based on data from metabolomic studies. In this study, enrichment analysis was used to identify important metabolic pathways that are significantly enriched in the urine samples of pregnant women with PE compared to control samples. The results of the enrichment analysis are shown graphically in Figure 3. The graph shows the enrichment of metabolic sets, with two major metabolic pathways identified as follows: sphingolipid metabolism and sulfate/sulfite metabolism.

Sphingolipid metabolism stands out with an enrichment ratio higher than 4, highlighting it as the dominant metabolic pathway associated with the analyzed samples and significant metabolites. This level of enrichment suggests that alterations in sphingolipid metabolism may be a key factor in the pathophysiological processes within the placenta associated with PE, which is also confirmed by a very low *p*-value (less than 0.02). Although less pronounced, sulfate and sulfite metabolism also show a significant enrichment, with an enrichment ratio of about 3.5 and *p*-values between 0.04 and 0.06. Although the statistical significance is lower compared to sphingolipid metabolism, the results still indicate a possible link between this metabolic pathway and PE.

The metabolite set enrichment analysis, shown in Figure 3, not only highlights the major metabolic pathways but also emphasizes the cellular locations where these pathways are predominantly active. This cellular localization highlights specific regions within the cell that may be particularly affected by the pathophysiology of PE and provides insight into how oxidative stress and metabolic dysfunction manifest in both the intracellular and extracellular spaces.

Among the most enriched metabolic pathways are those associated with the endoplasmic reticulum (ER) and the extracellular space. These results suggest that the ER, an important site for protein folding, lipid metabolism and cellular stress responses, is significantly involved in the metabolic disturbances seen in PE.

## 3. Discussion

The identification of hexadecanal as a statistically significant compound associated with PE underscores its potential as a biomarker for this complex pregnancy disorder [19,20]. This finding is particularly important as the role of oxidative stress and lipid peroxidation in the pathophysiology of PE is well established. Hexadecanal, a product of lipid peroxidation, may serve as a key indicator of oxidation-induced placental dysfunction, which is a hallmark of PE [11].

Previous studies have emphasized the importance of lipid-derived aldehydes, such as 4-hydroxy-2-nonenal (HNE) and malondialdehyde (MDA), in the context of oxidative stress and carbonyl stress, both of which are closely associated with placental aging and damage in PE. While hexadecanal itself has been less extensively studied compared to these aldehydes, its formation during sphingolipid metabolism makes it a promising candidate for further investigation. The role of hexadecanal in oxidative damage is supported by its ability to form protein adducts and alter membrane stability, similar to other reactive aldehydes [12,21,22].

Hexadecanal is formed as a metabolic by-product of the degradation of sphingosine-1-phosphate (S1P) by S1P lyase, a critical enzyme in sphingolipid metabolism [23]. Sphingolipids are important bioactive molecules that regulate various cellular processes, including apoptosis, proliferation and inflammatory processes, which are of great importance for the development of PE [24,25]. The disruption of sphingolipid metabolism in PE, particularly through the formation of reactive aldehydes such as hexadecanal, may contribute to endothelial dysfunction and inflammatory responses, both of which are key features of the disease [19].

In addition, oxidative stress induced by poor placentation and repeated ischemia–reperfusion injury is a known mechanism for the progression of PE [5,20,26]. The increased production of reactive oxygen species (ROS) leads to the peroxidation of polyunsaturated fatty acids, which in turn produces a number of reactive aldehydes, including hexadecanal. As Velegrakis et al. have shown, oxidative stress plays a central role in placental dysfunction, particularly through the induction of apoptosis and inflammation [4]. The involvement of hexadecanal in lipid peroxidation places it at the interface of these pathological processes, suggesting that it may be an important biomarker for the detection of early signs of oxidative damage in the placenta.

The two-stage model of PE, as outlined by Dimitriadis et al., underpins the relevance of hexadecanal as a potential biomarker [1]. Stage 1 is characterized by placental dysfunction due to defective trophoblast invasion and oxidative stress, while stage 2 is characterized by maternal symptoms such as hypertension and proteinuria. Hexadecanal may play a key role in stage 1 by reflecting early oxidative damage and lipid peroxidation in the placenta, which could make it a valuable biomarker for early diagnosis or risk stratification in pregnant women.

In addition, the enrichment analysis revealed that sulfate/sulfite metabolism plays an important role in PE. The cellular mechanisms responsible for maintaining redox homeostasis and antioxidant defense systems are highly dependent on the regulated reactivity of sulfur atoms, especially those derived from the amino acids cysteine and methionine. Under oxidative stress, cells strive to generate reducing agents to counteract the damaging effects of ROS. Sulfur-containing molecules play a crucial role in this process as they are involved in antioxidant metabolic pathways. As Miller et al. pointed out, the reactivity of sulfur in these molecules is essential for neutralizing oxidative damage [27].

The fact that oxidative stress has been identified in several studies as the main cause of PE reinforces the need for biomarkers that reflect this pathological process. [27,28]. Current efforts to screen for PE biomarkers, such as the sFlt-1/PlGF ratio, have proven useful but not definitive, and hexadecanal may complement these angiogenic markers by providing insights into the oxidative stress component of PE [29,30].

While these results are promising, it is important to consider potential limitations and areas for future research. Although hexadecanal has emerged as a significant feature in this study, larger studies with more diverse populations are needed to confirm its diagnostic utility. Furthermore, its specificity and sensitivity as a clinical biomarker need to be further investigated, especially in comparison to established markers such as the sFlt-1/PlGF ratio. In addition, a deeper understanding of the mechanistic role of hexadecanal in placental dysfunction, particularly its interactions with lipid peroxidation pathways and sphingolipid metabolism, is crucial for further development of its potential use in clinical settings.

## 4. Materials and Methods

### 4.1. Sample Preparation

Urine samples were collected from two groups of participants: 45 samples from pregnant women diagnosed with PE and 46 control samples. After collection, the urine samples were immediately frozen and stored at −80 °C until analysis. Prior to analysis, all samples were thawed at room temperature and prepared for SPME to isolate volatile compounds.

For each sample, 1 mL of urine was transferred into a 20 mL headspace vial, to which 0.2 mL of 2.5 M sulfuric acid was added to acidify the sample, following the method described by Aggarwal et al. [16]. After vortexing, the vials were sealed with polytetrafluoroethylene (PTFE)-coated silicone septa.

The sealed vials were then incubated at 60 °C for 30 min to allow volatile compounds to equilibrate in the headspace above the urine. Following the incubation period, an SPME fiber (50/30 μm divinylbenzene/carboxen/polydimethylsiloxane, DVB/CAR/PDMS, Supelco) was exposed to the headspace for 20 min in static mode to capture the volatile metabolites. After extraction, the fiber was withdrawn and immediately introduced into the GC-MS inlet for thermal desorption.

### 4.2. GS-MS Analysis

Following sample preparation, GC-MS was employed to identify volatile compounds isolated from the urine samples. The samples were analyzed using an Agilent 8890 GC system coupled with a 7000D triple quadrupole mass spectrometer (GC/TQ, Agilent Technologies, Santa Clara, CA, USA). The separation of volatile compounds was performed using an HP-5 MS capillary column (30 m × 0.25 mm × 0.25 µm, Agilent Technologies, Agilent Technologies, Santa Clara, CA, USA). The SPME fiber was inserted into the GC inlet for desorption at 220 °C for 5 min. The GC oven temperature program was set to an initial temperature of 40 °C, held for 2 min, and then increased at a rate of 5 °C/min to a final temperature of 220 °C, which was maintained for 4 min, resulting in a total run time of 42 min.

A solvent delay of 4 min was applied to prevent solvent peaks from entering the mass spectrometer. The mass spectrometer operated in positive electron ionization mode (EI+), scanning for a mass range of 10–300 *m/z*. The scan time was set to 0.1 s, with a resolution of 1000 at full-width half maximum (FWHM). Helium was used as the carrier gas at a flow rate of 1 mL/min.

### 4.3. Data Processing

After GC-MS analysis, data were normalized to minimize systematic bias and reduce technical variation to ensure accurate identification of metabolomic changes. All raw mass spectrometry data were processed using MS-DIAL software ver. 4.9., which enabled deconvolution, denoising, smoothing, peak identification, peak alignment and peak filtering [31,32].

Unknown metabolites were identified by comparison with the NIST library, and further validation and biological interpretation were performed using the Human Metabolome Database (HMDB). After identification, the data matrix was uploaded to the MetaboAnalyst 6.0 platform for multivariate statistical analysis [32]. The data matrix contained 91 samples with 107 peaks (*m/z* and retention time). The two groups were defined as follows: the preeclampsia (PE) group, consisting of 45 samples, and the control group, consisting of 46 samples.

Before statistical analyses, the data were standardized to the median. Three univariate statistical tests were performed to assess the differences between the groups: fold-change analysis, *t*-tests and volcano plots. The significance threshold for the *t*-test was set at *p* < 0.05 and the fold change (FC) threshold was set at 2.

Following the initial univariate analyses, ROC curve analysis was employed to evaluate the diagnostic potential of the identified biomarker.

In addition, the identified potential biomarkers were subjected to enrichment and pathway analysis using the MetaboAnalyst 6.0 platform. Pathways with an impact score of more than 0.10 were classified as significant and potentially relevant to the pathophysiology of PE.

## 5. Conclusions

In conclusion, the identification of hexadecanal as a significant compound in PE studies, in conjunction with its involvement in sphingolipid metabolism and oxidative stress, underscores its potential as a biomarker for early detection and possible monitoring of PE. This study highlights the ability of hexadecanal to indicate early oxidative damage in the placenta, which plays a key role in the pathophysiology of PE. Its diagnostic potential demonstrated by ROC analysis suggests that hexadecanal may complement existing angiogenic markers such as the sFlt-1/PlGF ratio and allow for a more comprehensive assessment of both angiogenic and oxidative signaling pathways in PE. Future research should further investigate the mechanistic role of hexadecanal in placental dysfunction, particularly its interaction with lipid peroxidation pathways, and evaluate its clinical utility in broader cohorts. The inclusion of hexadecanal in biomarker panels could improve early detection and risk stratification for women at high risk of PE and ultimately improve clinical outcomes for both mothers and their infants.

## Figures and Tables

**Table 1:** Baseline clinical characteristics of the study participants.

	Preeclampsia (N = 45)	Controls (N = 46)	*p*
Age, years			
Mean (SD)	32.1 (4.4)	32.6 (4.8)	0.556
95% CI	30.7–33.4	31.2–34.1	
Parity			
Median (IQR, 25th–75th)	1 (1, 1–2)	2 (1, 1–2)	0.003 *
95% CI	1.2–1.7	1.6–2.1	
Gestational age, weeks			
Median (IQR, 25th–75th)	36 (9, 29–38)	37 (4.5, 33.5–38)	0.202
95% CI	32.5–36.5	34.4–37.7	
BMI, kg/m^2^			
Median (IQR, 25th–75th)	26.35 (7.33, 23.88–31.21)	24.51 (5.21, 21.84–27.04)	0.016 *
95% CI	26.1–29.4	23.5–26.1	
Pregnancy weight gain, kg			
Median (IQR, 25th–75th)	13 (10, 7–17)	13 (6, 11–17)	0.263
95% CI	10.5–13.8	12.1–15.3	

BMI—body mass index. Data presented with mean (SD) or median (IQR, 25th–75th percentile), according to distribution and analyzed using Student’s *t*-test or Mann–Whitney *U* test. Gestational age pertaining to urine draw at PE onset or hospitalization of control group. * *p* < 0.05.

**Table 2:** Identified volatile metabolites in urine samples from preeclampsia and control groups using HS-SPME-GC-MS analysis.

No	RI	RT (min)	Compound
1	882	4.90	3,3-dimethylcyclohexanol
10	886	6.86	4-heptanone
16	902	7.46	2-heptanone
18	927	8.18	4-hydroxyphenylacetaldehyde-oxime
20	969	9.51	benzaldehyde
24	981	9.94	2-methyl-5-(methylthio)-furan
27	981	10.3	phenol
34	1030	11.52	1,2,3,4-tetramethyl-benzene (Prehnitol)
39	1053	12.23	tetrahydro-2,2-dimethyl-5-(1-methyl-1-propenyl)-furan
42	1077	13.01	2,6-dimethyl-7-octen-2-ol
45	1081	13.17	benzenemethanol
48	1084	13.26	1-phenylethanol
49	1092	13.53	butenylbenzene
50	1099	13.8	tetrahydrolinalool
52	1120	14.39	2,5-dihydroxybenzaldehyde
59	1176	16.04	menthol
60	1184	16.31	1,3,5-undecatriene
64	1193	16.60	1,2,3,4,tetrahydro-1,5,7-trimethylnapthalene
65	1205	16.97	decanal
66	1212	17.15	1,2,3,4-tetrahydro-1,1,6-trimethyl-naphtalene (alpha-ionene)
67	1215	17.21	4,7-dimethyl-benzofuran
81	1281	19.07	vitispirane
86	1300	19.62	2,6,10,10-tetramethyl-1-oxa-spiro[4.5]dec-6-ene
87	1303	19.7	undecanal
88	1330	20.39	3-formyl-N-methyl-9-[phenylethynyl]dibenzo[2,3-a:5,6-a′] (1,4)-thiazine
89	1356	21.07	1,2-dihydro-1,1,6-trimethylnaphthalene
92	1387	21.89	beta-damascenone
95	1487	24.4	4-(2,6,6-trimethyl-1,3-cyclohexadien-1-yl)-2-butanone
107	1817	31.8	hexadecanal

RI—RI-Kovats retention index, RT—retention time.

**Table 3:** Significant volatile metabolites identified by fold change and statistical analysis between the preeclampsia and control groups.

Compound	*p*-Value	FC (PE/C)
2-methyl-5-(methylthio)-furan	0.038	0.72
benzenemethanol	0.042	0.68
1-phenylethanol	0.014	0.61
1,2,3,4-tetrahydro-1,1,6-trimethyl-naphtalene	0.043	0.23
vitispirane	0.003	0.45
1,2-dihydro-1,1,6-trimethylnaphthalene	0.029	0.66
hexadecanal	0.042	2.75

## Data Availability

The datasets used in the current study are available from the corresponding author upon reasonable request.
